# Routes to rupture and folding of graphene on rough 6H-SiC(0001) and their identification

**DOI:** 10.3762/bjnano.4.69

**Published:** 2013-10-07

**Authors:** M Temmen, O Ochedowski, B Kleine Bussmann, M Schleberger, M Reichling, T R J Bollmann

**Affiliations:** 1 Fachbereich Physik, Universität Osnabrück, Barbarastraße 7, 49076 Osnabrück, Germany; 2 Fakultät für Physik and CeNIDE, Universität Duisburg-Essen, 47048 Duisburg, Germany

**Keywords:** graphene, Kelvin probe force microscopy (KPFM), local contact potential difference (LCPD), non-contact atomic force microscopy (NC-AFM), SiC

## Abstract

Twisted few layer graphene (FLG) is highly attractive from an application point of view, due to its extraordinary electronic properties. In order to study its properties, we demonstrate and discuss three different routes to in situ create and identify (twisted) FLG. Single layer graphene (SLG) sheets mechanically exfoliated under ambient conditions on 6H-SiC(0001) are modified by (i) swift heavy ion (SHI) irradiation, (ii) by a force microscope tip and (iii) by severe heating. The resulting surface topography and the surface potential are investigated with non-contact atomic force microscopy (NC-AFM) and Kelvin probe force microscopy (KPFM). SHI irradiation results in rupture of the SLG sheets, thereby creating foldings and bilayer graphene (BLG). Applying the other modification methods creates enlarged (twisted) graphene foldings that show rupture along preferential edges of zigzag and armchair type. Peeling at a folding over an edge different from a low index crystallographic direction can result in twisted BLG, showing a similar height as Bernal (or AA-stacked) BLG in NC-AFM images. The rotational stacking can be identified by a significant contrast in the local contact potential difference (LCPD) measured by KPFM.

## Introduction

Since its discovery in 2004 [1], graphene, the 2D crystal with a honeycomb lattice of sp^2^-bonded carbon atoms, has been shown to have unique properties such as high mechanical strength and elasticity, a very high electrical and thermal conductivity, the impermeability to gases, and many others [2]. All of them make it highly attractive for numerous applications, and a most promising candidate for advanced microelectronics technology [3], in which especially bilayer graphene (BLG) is of interest, as its band gap can be tuned [4]. Although the electronic properties of AB-stacked (Bernal) BLG is of special interest due to its tunable bandgap, rotationally stacked or twisted BLG is more attractive from an application point of view due to its angle-dependent electronic properties [4–10]. Twisted few layer graphene (FLG) exhibits electronic properties ranging from Dirac cones found for single layer graphene (SLG) for rotation angles over 15° where the layers are effectively decoupled, to a Fermi velocity renormalization for smaller rotation angles [8–11]. For very small rotation angles, θ ≤ 2°, the electronic properties are found to be coupled to the resulting moiré spots for twisted BLG [10].

In order to study these properties experimentally, (few layer) graphene can be produced in various ways. The growth of graphene on metals followed by transfer to another substrate as well as epitaxial growth on SiC, both have a potential for mass production if technological shortcomings can be overcome. However, exfoliation from graphite still results in graphene flakes of highest quality [1–2], which then can be modified in situ to create (twisted) FLG. In comparison to the well known epitaxial growth of graphene on SiC [12–14], here we study mechanically exfoliated graphene on 6H-SiC(0001) to produce large sheets of high quality. Defects are first created by swift heavy ions (SHI). The unique controllability of SHI irradiation, by tuning the incident angle with respect to the crystallographic directions of graphene as well as the range and the energy loss mechanism, makes it a viable route for one-dimensional controlled defect creation relevant for future applications [15–16]. By the use of ions having kinetic energies in the range of 100 MeV impinging under grazing incidence, it has already been shown that SLG flakes can be ruptured in a controlled process by highly localized energy deposition [15]. This results in foldings that are BLG sheets produced in the vicinity of the ion track. Foldings can also be produced by line scanning the sample with an AFM tip in the contact mode, in which the tip forces are capable of rupturing the sheet [17]. Here, we report that also severe heating is able to create foldings on SLG, deposited under ambient conditions. The modification method making use of SHI irradiation is further on referred to as method (i), contacting and line scanning, as method (ii), and severe heating, as method (iii), respectively. The combination of the latter two is further on referred to as *post-preparation treatment*. Note that in some cases the origin of the foldings can not be uniquely identified as method (ii) or (iii) as AFM imaging involves strong tip–sample interactions.

Rupture and folding result in a system where we can study and compare properties of graphene in several thicknesses, sheet orientations, edges [18–19] and stackings. To discriminate the different BLG stackings, we investigate the topography by non-contact atomic force microscopy (NC-AFM) combined with measuring the local contact potential differences (LCPD) using Kelvin probe force microscopy (KPFM).

## Experimental

Graphene is exfoliated from a HOPG crystal (Momentive Performance Materials, Columbus, OH, USA) under ambient conditions on an as delivered Si-rich 6H-SiC(0001) substrate (Pam-XIAMEN, Xiamen, China) applying a well known recipe [1]. After preparation, the sample is taken in ambient atmosphere to the IRRSUD beamline of the Grand Accélérateur National d’Ions Lourds GANIL (Caen, France) for SHI irradiation with 81 MeV Ta^24+^ ions under 1.5° grazing incidence. The ion fluence is adjusted to 5–10 ions/μm^2^.

Irradiated samples are initially inspected by tapping mode atomic force microscopy performed under ambient conditions using a Dimension 3100 AFM (Veeco Metrology, Santa Barbara, CA, USA) and NCHR cantilevers (Nanosensors, Neuchatel, Switzerland). NC-AFM images are obtained using a well characterized [20–22] UHV 750 NC-AFM system (RHK Technology, Troy, MI, USA) in an ultra-high vacuum chamber with a base pressure well below 5 × 10^−11^ mbar. Force sensors used are NCH Si cantilevers (Nanosensors, Neuchatel, Switzerland) with the exception of the measurements presented in Figure 7a, in which a Vistaprobe T-300 cantilever (Vistaprobe, Phoenix, AZ, USA) was used. Both cantilevers have a fundamental eigenfrequency *f*_0_ 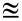
300 kHz. To obtain correct height measurements, KPFM [23–24] imaging is performed simultaneously by applying an AC voltage of 1 V amplitude at a frequency of 1.2 kHz added to the DC bias regulated to minimize electrostatic forces. To remove volatile surface contaminants that can significantly influence LCPD measurements [25], the sample is heated in UHV to 500 K prior to measurements. All images in this paper are presented without filtering or smoothing. The topographic images are compensated for piezo creep and drift as well as for scanner bow using common plane subtraction and (facet) leveling algorithms of the Gwyddion software package [26].

## Results and Discussion

### Graphene flake characterization

**Figure 1:**
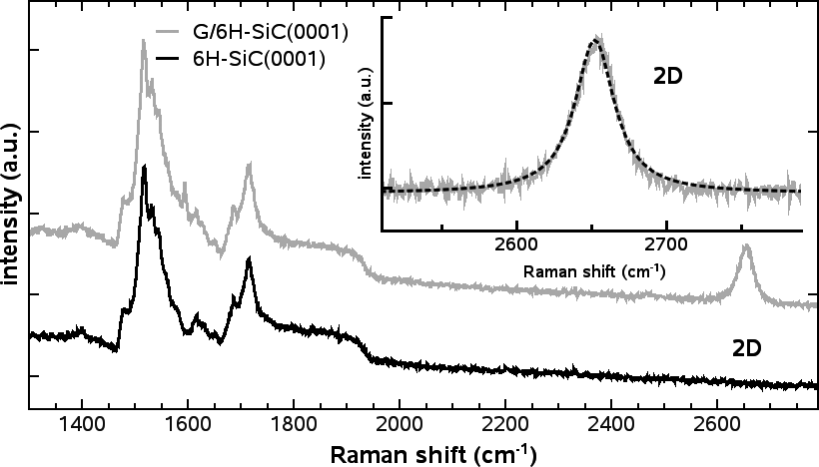
Micro-Raman spectrum for both, the substrate and the SLG flake introduced in Figure 3. The inset shows the 2D Raman peak and its Lorentzian fit.

After mechanically exfoliating graphene on the 6H-SiC(0001) substrate, a flake is selected that has a width of 2–3 μm and a length of ≈40 μm. The flake shows straight parallel edges and the apparent thickness of the SLG flake is 0.7 ± 0.4 nm. Since a thickness measurement of SLG flakes is not straightforward and thickness values reported in literature range from 0.35 nm to ≈1 nm [1,27–29], we use a LabRAM HR micro-Raman spectrometer (Horiba, Kyoto, Japan) to confirm the identity as SLG using its 2D band [30–31]. Figure 1 shows the Raman spectrum measured for the 6H-SiC(0001) substrate and the SLG covered substrate. The sharpness and symmetry of the 2D band around 2650 cm^−1^ combined with the quality of the single Lorentzian fit are characteristic for the presence of SLG [30–31].

**Figure 2:**
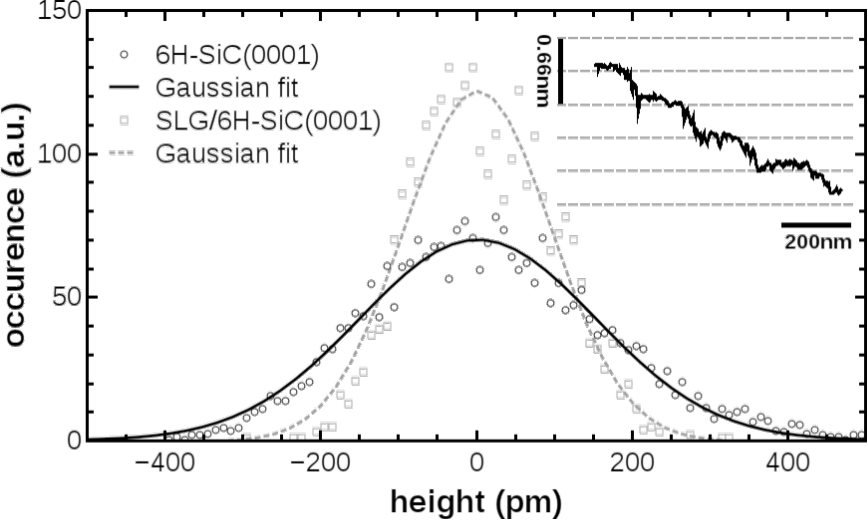
Roughness analysis of NC-AFM images taken on a single terrace within 70 × 70 nm^2^ on the 6H-SiC(0001) substrate (standard deviation σ = 304 pm) and on the SLG (σ = 189 pm). The inset shows a line profile taken on the SLG flake perpendicular to substrate step edges measured by Kelvin-compensated NC-AFM.

Mechanically exfoliated graphene is known to adapt to its substrate like a carpet [32]. The Kelvin-compensated NC-AFM topography measurements taken on SLG reflect the substrate step structure with its bilayer step height of 0.33 ± 0.10 nm [13]. A representative line profile is shown in the inset of Figure 2. To determine whether the roughness on the SLG reflects the contours of the underlying substrate, we determine the height distribution on both, the bare substrate and the graphene covered substrate in 70 × 70 nm^2^ areas free of steps. The roughness can be characterized by the standard deviation σ of the height distribution [32] shown in Figure 2. For the exfoliated SLG sheet we find a roughness that is about 60% of the substrate roughness, indicating that graphene adapts to its underlying substrate closely but removes part of its roughness.

### Methods for rupture and folding of graphene

**Figure 3:**
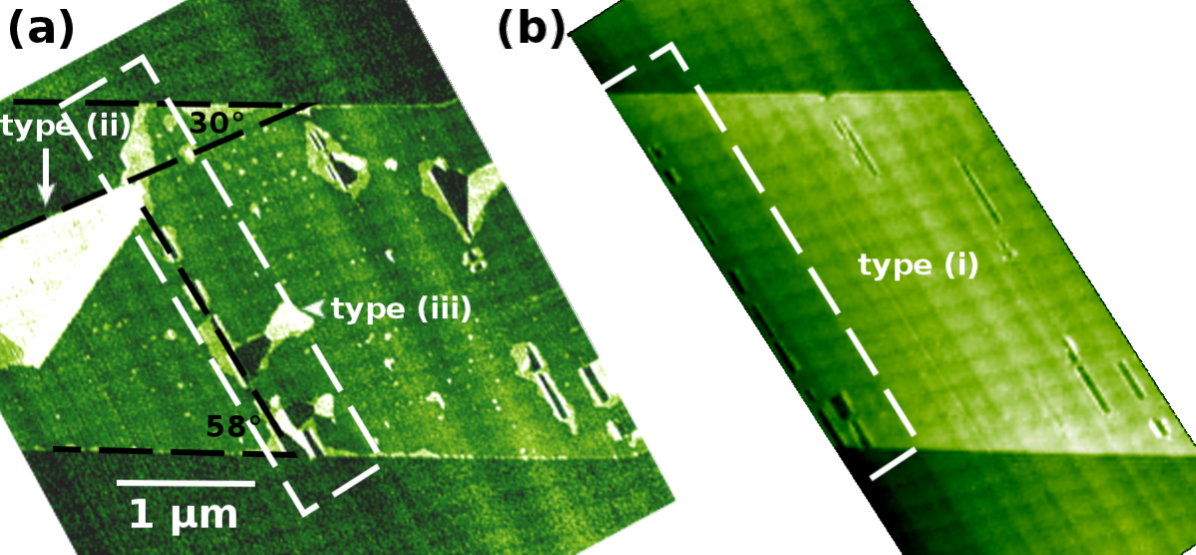
Tapping mode AFM image (a) of an exfoliated SLG flake on 6H-SiC(0001) showing examples of rupture and folding by the use of SHI, type (i), modification by AFM tip contacting and linescanning (ii), and severe heating (iii). Tapping mode AFM image (b) showing the modifications of SLG solely due to SHI irradiation, type (i), namely foldings aligned with the ion track. The dashed box in Figure 3(a) exhibiting modifications of type (ii) and (iii) added later, corresponds to the dashed box in Figure 3(b) prior to post-preparation treatment.

To create FLG including layers with twisted stacking, the exfoliated graphene is exposed to SHI irradiation (i) followed by a combination of line scanning the sample with an AFM tip (ii) and severe heating (iii). The tapping mode AFM survey image shown in Figure 3a exhibits a representative part of the flake comprising all phenomena discussed in this paper, rupture and folding of various origin, labeled by (i), (ii) and (iii), respectively. The tapping mode AFM survey image shown in Figure 3b, has been taken right after SHI irradiation where the SLG shows foldings at an angle of 58.0 ± 1.2° with respect to the flakes edge. The properties of such foldings related to the ion track have been described in more detail elsewhere [15]. Defects created by method (i) can be used as a seed for further rupture created by methods (ii) and (iii). The dimensions of the tip-induced foldings (ii) are similar to the ones reported in literature and can be created without defects of type (i) [17]. Severe heating results in the opening at locations of existing rupture, creating type (iii) structures. For this type of modification, the interfacial layer residing between the substrate and the SLG flake due to its exfoliation in ambient, is anticipated to play a major role. Thin water films resulting from exfoliation in ambient have been recognized in literature as an important feature determining the sheet properties [33–39]. The structures of type (iii) might act as a pressure release for heated water confined at the interface. The dashed box in Figure 3a shows the same region as the dashed box in Figure 3b after heating to 700 K. The onset of the heating effect is found at about 500 K.

### Discriminating graphene stackings by their surface potential

**Figure 4:**
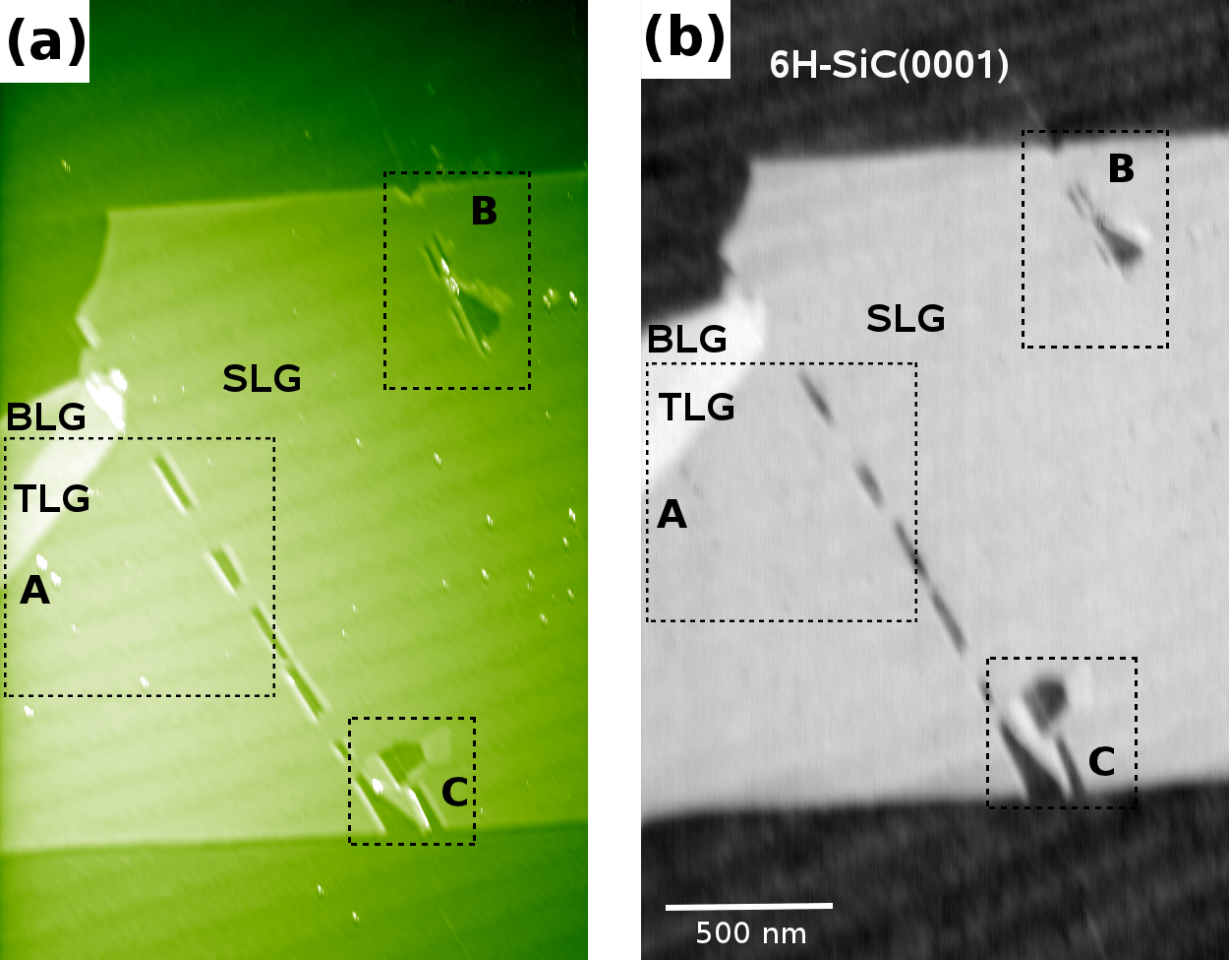
NC-AFM image (a) of an exfoliated SLG flake on 6H-SiC(0001), irradiated with SHI and subjected to post-preparation treatment, and its corresponding LCPD image (b). Folding structures, which result from post-preparation treatment labeled by A, B and C are analyzed in detail in Figure 5, Figure 6 and Figure 7.

Next, we investigate the graphene layer, its rupture, foldings and stacking in more detail by Kelvin compensated NC-AFM imaging, demonstrating the identification of (twisted) FLG. To study the properties of rupture and foldings in the SLG originating from treatment with methods (i), (ii) and (iii) in detail, we analyze images taken in the areas A, B and C marked by the boxes in Figure 4.

**Figure 5:**
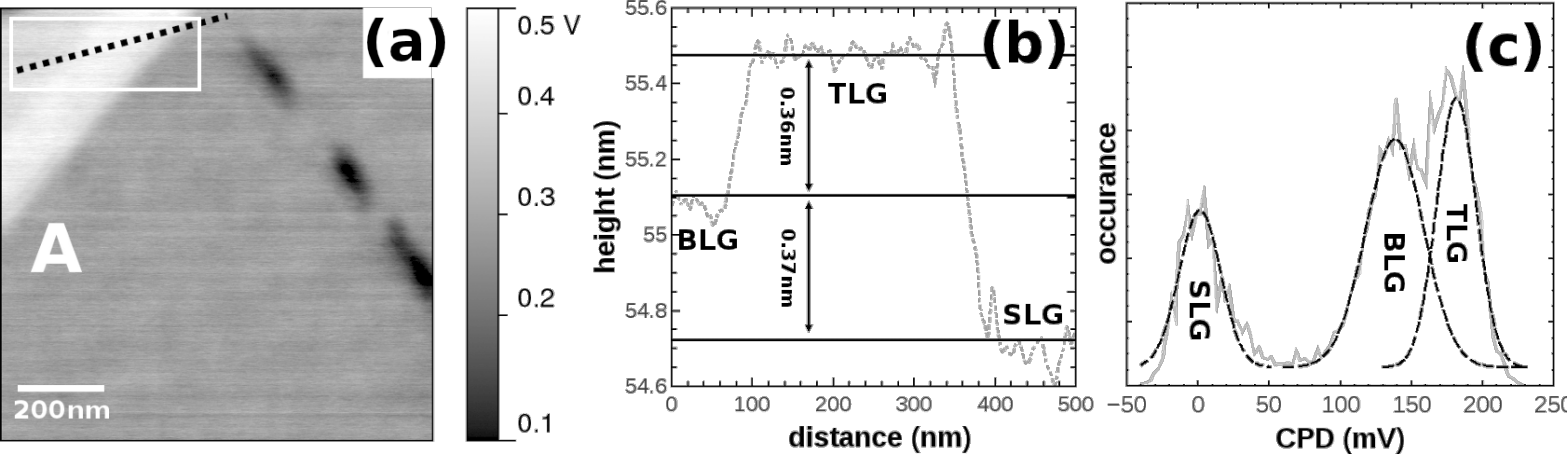
(a) LCPD image of the folding marked by A in Figure 4. (b) The profile taken in the corresponding NC-AFM image reveals the height of BLG and TLG in agreement with the interlayer distance (marked by horizontal full lines) found for graphite stacking. (c) From the CPD histogram performed in the square marked in Figure 5a, the LCPD between SLG and BLG (137 ± 40 meV) and BLG and TLG (43 ± 27 meV) can be identified by normal distributions, in which the error is one standard deviation (σ).

**Figure 6:**
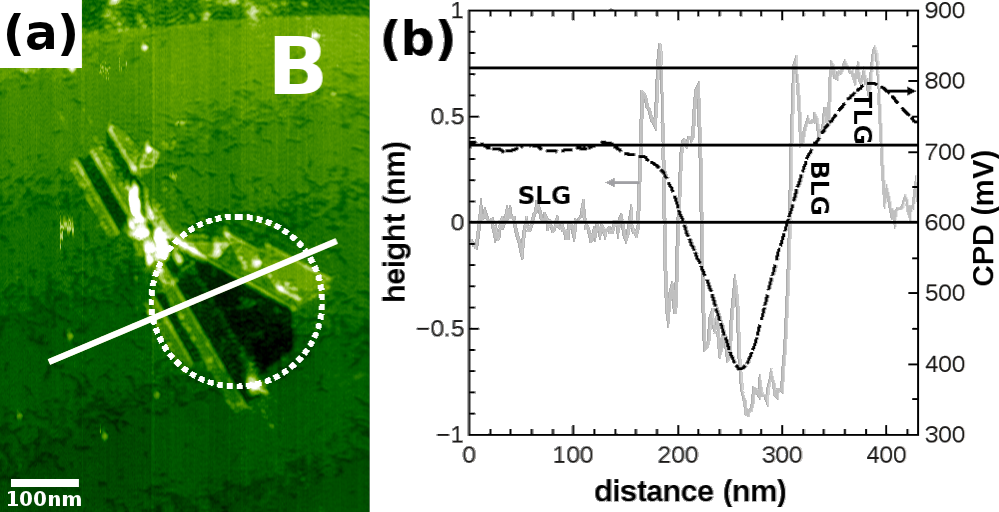
(a) NC-AFM image of the region marked by B in Figure 4, where post-preparation treatment resulted in the folding encircled by the dashed line. Line profiles (b) reveal the height (solid gray line) of BLG (limited accuracy due to areal size and spatial resolution) and TLG (+0.72 nm with respect to SLG) in agreement with the interlayer distance (marked by horizontal full lines) found for graphite stacking. From the LCPD line profile (dashed black line), the LCPD of the different foldings can not be discriminated due to the limited spatial resolution.

The folding marked by A in Figure 4 is a typical example of a type (ii) folding, in which the folding over 30° with respect to the SLG flakes edge (see also Figure 3), results in large Bernal-stacked (or energetically unfavorable AA-stacked) areas of BLG and trilayer graphene (TLG). Figure 5a shows a detailed KPFM image of region A, the corresponding height profile in Figure 5b, and CPD histogram in Figure 5c performed in the white square shown in Figure 5a, from which the LCPD of BLG and TLG with respect to SLG can be determined. The LCPD between SLG and BLG is found to be 137 mV, which is in agreement with the LCPD found in a previous study [16,40] as well as for epitaxially grown SLG and BLG [12]. Between BLG and TLG, the LCPD is determined to be 43 mV. From the line profile in the topography, the interlayer distance between SLG and BLG (0.37 ± 0.10 nm) and BLG and TLG (0.36 ± 0.10 nm) are determined, in agreement with the interlayer distance found for graphite stacking [41–43]. A similar result is found for the folding marked by B, where the respective image is shown in Figure 6.

Figure 6a shows a detailed topographic image, in which the folding structure that results from SHI irradiation and post-preparation treatment is encircled. The edges of folding structures have angles of 30°, 90° and 120° with respect to the SLG flake edges reflecting the six-fold symmetry of the graphene lattice combined with the two most stable edge configurations, namely zigzag and armchair [18–19]. From the line profile (grey dashed line) in Figure 6b, the interlayer distance between SLG and TLG (0.72 nm) is determined, again in agreement with the interlayer distance found for graphite stacking [41–43]. For this folding, the areas for BLG and TLG are too small in size and too close to each other to be spatially resolved by KPFM. Therefore, the foldings can not be discriminated in the LCPD line profile (black full line) shown in Figure 6b.

**Figure 7:**
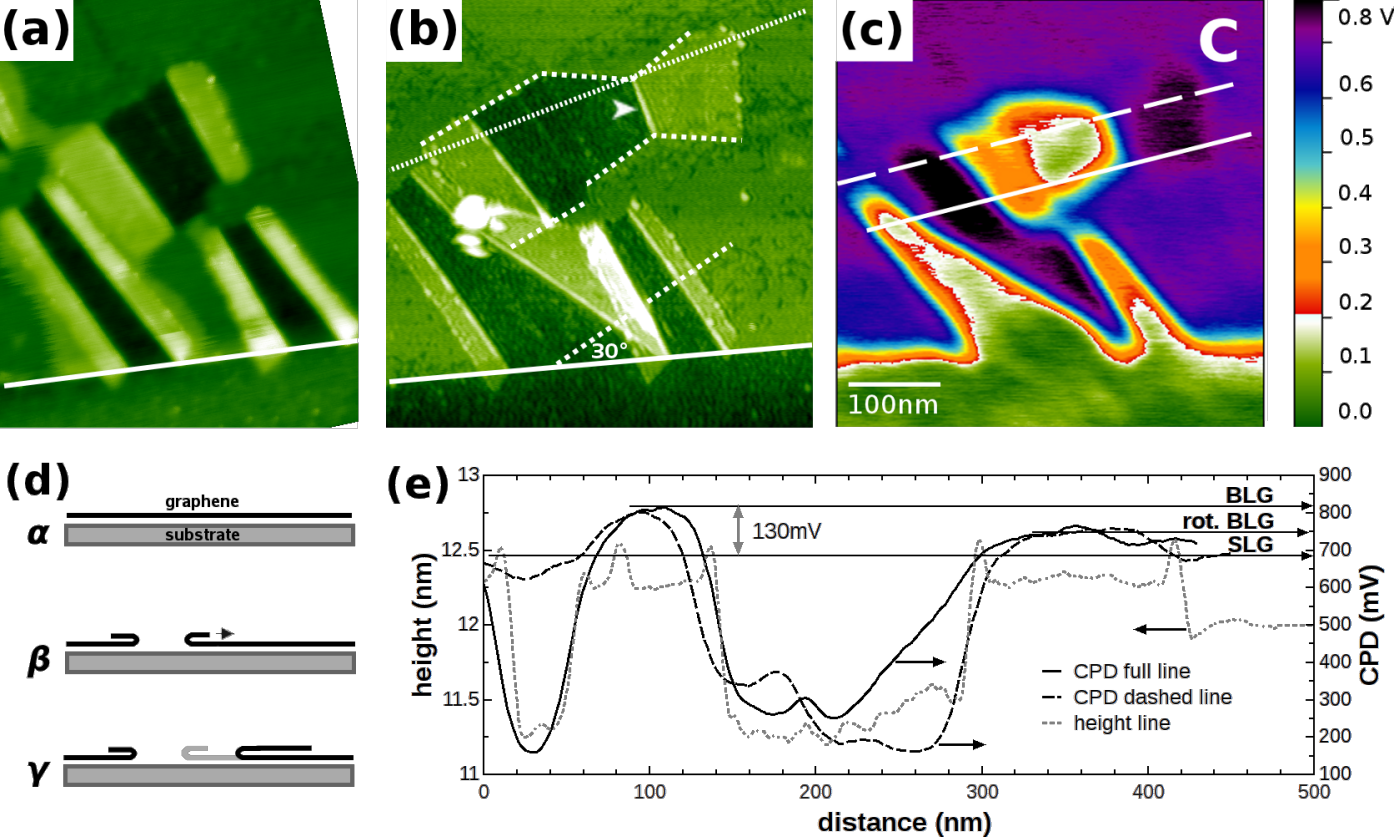
NC-AFM image of the region marked C in Figure 4 with foldings due to SHI impact on the left and right of the ion track prior to (a) and after (b) post-preparation treatment. (b) Large folding created by post-preparation treatment, where the identified edges, zigzag and armchair, are labeled by (white) full and dashed lines and make angles of 30° with respect to each other as exemplified at the bottom. (c) LCPD image taken at the same position but extracted from a different image. (d) Cartoons of the graphene exfoliated on the substrate (α), after SHI irradiation creating BLG (β), followed by applying post-preparation methods where the direction of peeling the folding on the right is marked by the arrow (γ). (e) Line profiles taken in frames (b) and (c) reveal the same height but a significant difference in the LCPD for the two foldings. The LCPD difference is attributed to the stacking difference as expected from the misalignment of the right folding with respect to the low index crystallographic directions of the SLG flake.

Figure 7a shows a detailed topographic image of region C right after SHI bombardment and slight heating, which results in foldings that are BLG sheets aligned with the ion track. Figure 7b shows the same region C after applying post-preparation treatment, which results in enlarged foldings left and right of the region already opened by the SHI impact. In contrast to the situation right after SHI irradiation, foldings appear ruptured along characteristic angles with respect to the flake edges. As can be seen from the comparison of Figure 7a and Figure 7b, the post-preparation treatment of a SLG sheet, which has been folded, can be interpreted as peeling the folding away from the region already opened by the SHI impact as illustrated in Figure 7d [44]. Due to the high elasticity of graphene, when peeling the folding by applying the post-preparation methods, the SLG detaches from the substrate and folds back onto itself, thereby extending the folding and enlarging the opened region. Peeling at an angle can result in a twisted BLG sheet with rupturing along preferential directions as demonstrated below.

To understand the role of the preferential directions of rupture, we start by identifying the edge of the large SLG flake, marked by the white line at the bottom of the image. The edge resulting from exfoliation is expected to have a preferential direction being either a zigzag or an armchair edge. Therefore, the vast majority of edges formed by rupture due to post-preparation treatment are found to be aligned at 30° and 60° with respect to the SLG flake edge, exemplified at the bottom of Figure 7b. Considering the symmetry of the graphene lattice, these ruptures can, therefore, be identified as either zigzag or armchair edges.

Assigning the SLG flake edge, being of either zigzag or armchair structure, the dashed lines enclosing an angle of 30° with the SLG flake edge, therefore, correspond to the other type edges. However, the edge over which the folding on the right took place, marked by the arrow in Figure 7b, has an angle of about 70° with respect to the SLG flake edges as a result of peeling under an angle by applying the post-preparation treatment. One might, therefore, expect a twisted or rotational stacking for the attached BLG. Note that, although peeling at a slight angle on the existing folding results in a twisted stacking, the (preferential) directions of rupture are unaffected by this. Comparing the height of this folding with the one on the left side of the SHI track as shown in Figure 7e does, however, not reveal any significant difference in height. The height found for both foldings is in agreement with the values found in Figure 5 and Figure 6. Within the error of several angstroms for the height measurement, we are unable to discriminate between the AA- and AB-stacking interlayer distance difference of 10% [42,45–47].

However, by the use of KPFM, one is able to clearly discriminate between different rotational stackings. In Figure 7c, we show the LCPD of the large BLG foldings on the left and right of the ion track. As a striking observation, we note that the two BLG foldings show a slight but significant difference in their LCPD. Care has to be taken by comparing the LCPD for the two foldings due to the influence of the substrate steps on the LCPD measurement. In this case, however, both foldings are on the same terrace and quite far away from any step edge. The line profiles drawn in Figure 7e, in the center and within close vicinity to the step edge, show identical behavior for the two foldings. The LCPD measured for the left (810 ± 5 mV) and for the right (754 ± 13 mV) folding, enables us to discriminate twisted BLG by ≈55 mV which is well above the variation in LCPD on a SLG or BLG sheet. The reduced potential of the twisted folding on the right is in between that of SLG and Bernal- (or energetically unfavourable AA-) stacked BLG.

## Conclusion

In summary, we demonstrate different routes to rupture and folding of SLG on a 6H-SiC(0001) substrate as well as experimental techniques to identify and discriminate the resulting (twisted) BLG and TLG structures. Rupture and folding of SLG on a 6H-SiC(0001) can be induced by scanning and contacting with an AFM tip and by severe heating resulting in foldings with (AA- or) Bernal-stacking and twisted stacking. SLG sheets rupture along preferential edges of zigzag and armchair type, even when peeling an existing folding is performed under an angle resulting in a twisted stacking. While the (AA- or) Bernal-stacked BLG and twisted BLG can not be discriminated by their height, they can clearly be distinguished by the difference in their LCPD.
